# Comparison of Modified Early Warning Score (MEWS), Simplified Acute Physiology Score II (SAPS II), Sequential Organ Failure Assessment (SOFA), and Acute Physiology and Chronic Health Evaluation II (APACHE II) for early prediction of septic shock in diabetic patients in Emergency Departments

**DOI:** 10.1186/s12873-024-01078-8

**Published:** 2024-09-04

**Authors:** Wijittra Liengswangwong, Ranchana Siriwannabhorn, Sittichok Leela-Amornsin, Chaiyaporn Yuksen, Pitsucha Sanguanwit, Chonthicha Duangsri, Nusara Kusonkhum, Parnthap Saelim

**Affiliations:** 1 Department of Emergency Medicine, Faculty of Medicine Ramathibodi Hospital, Mahidol University, Bangkok, Thailand; 2 Chakri Naruebodindra Medical Institute, Faculty of Medicine Ramathibodi Hospital, Mahidol University, 111 Moo 14, Bang Pla, Bang Phli, Samut Prakarn, Thailand

**Keywords:** Diabetes, Sepsis, Septic shock, Emergency department, Score

## Abstract

**Introduction:**

Sepsis is a severe medical condition that can be life-threatening. If sepsis progresses to septic shock, the mortality rate increases to around 40%, much higher than the 10% mortality observed in sepsis. Diabetes increases infection and sepsis risk, making management complex. Various scores of screening tools, such as Modified Early Warning Score (MEWS), Simplified Acute Physiology Score (SAPS II), Sequential Organ Failure Assessment Score (SOFA), and Acute Physiology and Chronic Health Evaluation (APACHE II), are used to predict the severity or mortality rate of disease. Our study aimed to compare the effectiveness and optimal cutoff points of these scores. We focused on the early prediction of septic shock in patients with diabetes in the Emergency Department (ED).

**Methods:**

We conducted a retrospective cohort study to collect data on patients with diabetes. We collected prediction factors and MEWS, SOFA, SAPS II and APACHE II scores to predict septic shock in these patients. We determined the optimal cutoff points for each score. Subsequently, we compared the identified scores with the gold standard for diagnosing septic shock by applying the Sepsis-3 criteria.

**Results:**

Systolic blood pressure (SBP), peripheral oxygen saturation (SpO2), Glasgow Coma Scale (GCS), pH, and lactate concentrations were significant predictors of septic shock (*p* < 0.001). The SOFA score performed well in predicting septic shock in patients with diabetes. The area under the receiver operating characteristics (ROC) curve for the SOFA score was 0.866 for detection within 48 h and 0.840 for detection after 2 h of admission to the ED, with the optimal cutoff score of ≥ 6.

**Conclusion:**

SBP, SpO2, GCS, pH, and lactate concentrations are crucial for the early prediction of septic shock in patients with diabetes. The SOFA score is a superior predictor for the onset of septic shock in patients with diabetes compared with MEWS, SAPS II, and APACHE II scores. Specifically, a cutoff of ≥ 6 in the SOFA score demonstrates high accuracy in predicting shock within 48 h post-ED visit and as early as 2 h after ED admission.

**Supplementary Information:**

The online version contains supplementary material available at 10.1186/s12873-024-01078-8.

## Introduction

Sepsis is a severe medical condition characterized by a systemic inflammatory response that frequently impacts various organ systems. [1, 2] In Thailand, the annual incidence of sepsis falls within the range of 75 to 150 cases per 100,000 individuals. Notably, sepsis is a significant contributor to mortality, accounting for 6% of all deaths, with 22% of these fatalities attributed to concurrent sepsis. [3] When sepsis progresses to the more critical state of septic shock, the mortality rate substantially escalates to approximately 40%, a stark contrast to the 10% mortality rate observed in cases of sepsis. [4]

Patients diagnosed with diabetes mellitus (DM) face an elevated susceptibility to infections and sepsis. [4] Furthermore, the management of sepsis in individuals with diabetes presents inherent complexities. [5] This issue is exacerbated by the growing prevalence of diabetes, as evidenced by data from Thailand’s National Health Examination Survey, which has documented a consistent annual rise in the incidence of DM cases. [6]

The Emergency Department (ED) typically serves as the initial point of contact for patients experiencing deteriorating health conditions. Timely recognition and management of sepsis within this critical setting are paramount, as delayed intervention can exacerbate the condition. Therefore, adhering to sepsis guidelines, which emphasize the importance of early fluid resuscitation and the prompt initiation of appropriate antibiotics within the first hour of presentation, is of utmost significance.

Numerous screening tools have been developed to aid in the identification of sepsis, including Systemic Inflammatory Response Syndrome (SIRS), Quick Sequential Organ Failure Assessment Score (qSOFA), Modified Early Warning Score (MEWS) [7], and the Ramathibodi Early Warning Score (REWS). [8] Additionally, scoring systems such as MEWS [9], Simplified Acute Physiology Score (SAPS II) [10], Sequential Organ Failure Assessment Score (SOFA) [2, 11], and Acute Physiology and Chronic Health Evaluation (APACHE II) [12, 13] are employed to predict the severity or mortality risk associated with the disease. Early recognition of sepsis severity prior to the onset of septic shock enables more vigilant monitoring and precise resuscitative efforts.

The principal objective of this investigation was to conduct a comparative assessment of the efficacy of MEWS, SAPS II, SOFA, and APACHE II scoring systems for the early prediction of septic shock in diabetes patients within the ED setting. Additionally, we sought to determine the optimal cutoff values for detecting septic shock. Our secondary aim entailed the analysis of data pertaining to patients presenting with concurrent diagnoses of diabetes mellitus and septic shock at Ramathibodi Hospital’s ED.

## Methods

### Study design and setting

We conducted a retrospective cohort study focusing on diagnostic accuracy within the ED of Ramathibodi Hospital in Bangkok, Thailand. This study received approval from the Human Research Ethics Committee at the Faculty of Medicine, Ramathibodi Hospital, Mahidol University. Ramathibodi Hospital is a tertiary care medical facility located in Bangkok, and the study was conducted following their ethical guidelines (COA.MURA2021/878).

### Study participants

We enrolled patients visiting Ramathibodi Hospital’s ED between 1 January 2017 and 31 December 2021. We included those aged > 18 years with DM. Inclusion criteria mandated a sepsis diagnosis according to the International Classification of Diseases, 10th Revision (ICD-10), within 48 h of their ED admission. The diagnostic criteria for sepsis were aligned with the latest international consensus guidelines, explicitly following the Sepsis-3 criteria.

Predetermined parameters were implemented and required the identification of a confirmed source of infection and the presence of at least one of the following criteria: qSOFA score of ≥ 2, SIRS score of ≥ 2, or REWS of ≥ 2. [8] Furthermore, septic shock was defined as the presence of a confirmed source of infection, accompanied by hypotension necessitating vasopressor use to maintain a Mean Arterial Pressure (MAP) ≥ 65 mmHg and adequate fluid resuscitation. [8] The exclusion criteria were as follows: presentation of sepsis after 48 h of triage in the ED, incomplete laboratory results, non-diabetic mellitus types 1 or 2, palliative care with the refusal of inotropes or an endotracheal tube in the ED, revisit of clinically unimproved cases, and a diagnosis of septic shock within 15 min after the ED visit.

### Data gathering

We collected data from 552 of 1,150 patients (66 patients had no data, and 532 had incomplete laboratory values). Data collection comprised a comprehensive set of variables, including patients’ demographic information, age, sex, comorbidities, glycemic control of DM patients categorized by the American Diabetes Association (ADA) guidelines for the year 2022 [14, 15], mode of transportation to the ED, initial vital signs in ED, Emergency Severity Index (ESI) triage, source of infection and initial laboratory findings such as white blood cell count, platelet count, serum creatinine, point-of-care glucose, electrolyte levels, albumin, bilirubin, arterial blood gas parameters, and serum lactate. Moreover, each patient’s SIRS, qSOFA, REWS, MEWS, SOFA, SAPS II, and APACHE II scores were recorded.

### Outcome

**Fig. 1:**
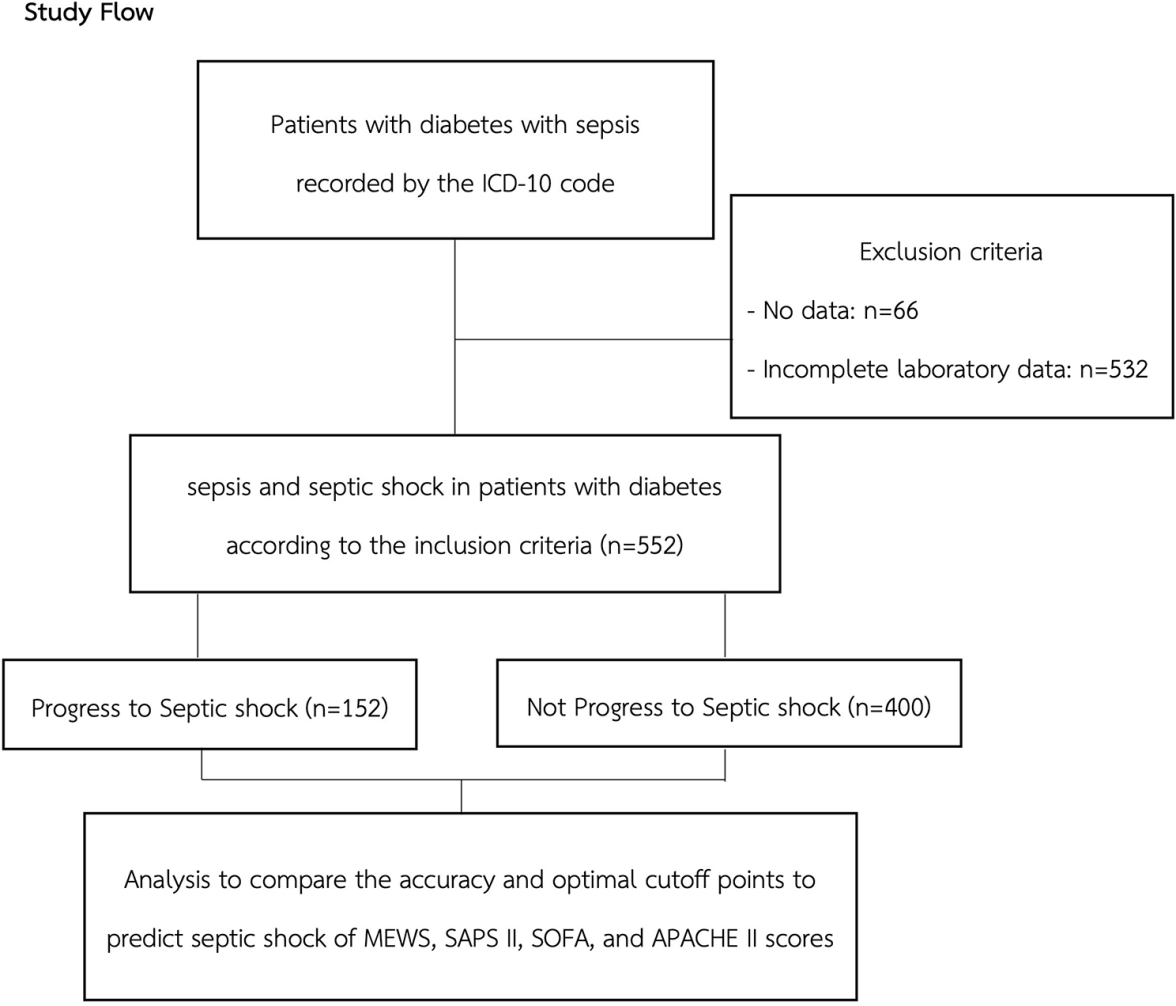
Study flow

The primary outcome of this study was the diagnosis of septic shock within a 48-hour timeframe. Secondary outcomes included the duration from triage to the progression of septic shock, admission to the intensive care unit (ICU), discharge from the ED, ED length of stay (LOS), overall hospital length of stay, 28-day mortality, in-hospital mortality, and the use of mechanical ventilation. The protocol is illustrated in Fig. 1.

### Sample size and statistical analyses

The sample size of this study was calculated by PM-sampsize statistical software on the basis of our pilot unpublished data. [16] We included 215 patients with diabetes. Using the C-statistic, the area under the curve (AUC) was 0.68 with three parameters (from qSOFA score). We assumed a 0.05 acceptable difference in the apparent and adjusted R-squared and a 0.05 margin of error in the estimation of intercept. When using the Events per Predictor Parameter (EPP), we assumed a prevalence of progression to septic shock of 0.10 by the STATA statistical program. The minimum sample size required for new model development was 693 with 70 events. Descriptive data are presented as the means with standard deviations (SD) or medians (interquartile ranges) for continuous variables and as the percentages for categorical variables. Chi-square and Wilcoxon’s rank-sum tests were used to compare categorical variables and continuous variables, respectively. We used the Youden index to determine a cutoff value for predicting progression to septic shock within 48 h for each score. Sensitivity, specificity, and positive likelihood ratio (LR+) were calculated to present the predictive performance separately for each score.

A discrimination result was considered satisfactory if the Receiver operating characteristic (ROC) curve area was > 0.7, with statistical significance denoted as *p* ≤ 0.05. The AUC, with a 95% CI, was used to evaluate the discrimination performance of each score. All tests were two-sided, and values with a *p* ≤ 0.05 were statistically significant. All data analysis was performed using Stata version 16 (StataCorp LLC, College Station, TX, USA).

## Results

A total of 1150 adult diabetic patients were presented to the ED of Ramathibodi Hospital and were diagnosed with sepsis between January 1, 2017, and December 31, 2021. Patients with missing data (*n* = 66) and incomplete laboratory results (*n* = 532) were excluded from the analysis. Consequently, 552 patients remained eligible and were included in the analysis (Fig. 1). Among these, 152 individuals (27.54%) progressed to septic shock within 48 h.

Table 1 shows the demographic and clinical characteristics of the patients. A higher incidence of septic shock was observed in males than in females, 77 (50.66%) vs. 137 (34.25%) *P* < 0.001. Patients with septic shock had significantly higher percentages of underlying diseases of myocardial infarction, Parkinson’s disease and dementia or Alzheimer’s disease than sepsis (49 (32.24%) vs. 78 (19.50%) *p* = 0.001, 11 (7.24%) vs. 10 (2.50%) *P* = 0.009, 15 (9.87%) vs. 11 (2.75%) *P* < 0.001, respectively). However, patients with septic shock had significantly lower percentages of underlying diseases of hypertension, metastatic cancer and old cerebrovascular accident (CVA) than sepsis group (76 (50%) vs. 271 (67.75%) *p* < 0.001, 22 (14.47%) vs. 116 (29.00%) *p* < 0.001, 47 (30.92%) vs. 191 (47.75%) *P* < 0.001, respectively).

**Table 1:** Baseline characteristics of patients with diabetes categorized by shock

Characteristics	All sepsis (*n* = 552)	Progress to Septic Shock (*n* = 152)	Not progress to septic shock (*n* = 400)	*p*-value
Age, years (mean ± SD)	75.84 ± 13.10	74.27 ± 13.25	76.43 ± 13.00	0.084
BMI > 30 kg/m^2^ (n, %)	59 (10.69)	19 (12.50)	4 (10.00)	0.396
Male sex (n, %)	214 (38.77)	77 (50.66)	137 (34.25)	< 0.001
HbA1C (mean ± SD)	6.90 ± 1.81	7.09 ± 2.13	6.83 ± 1.67	0.122
Poorly controlled DM (n, %)	90 (16.30)	32 (21.05)	58 (14.50)	0.063
EMS route (n, %)	32 (6.34)	11 (7.24)	21 (5.25)	0.386
Surgical service (n, %)	56 (10.14)	12 (7.89)	44 (11.00)	0.280
**Underlying disease**				
Hypertension (n, %)	347 (62.86)	76 (50)	271 (67.75)	< 0.001
Heart failure, NYHA IV (n, %)	53 (9.60)	17 (11.18)	36 (9.00)	0.437
Cardiovascular disease (n, %) - Myocardial infarction (n, %) - Arrhythmia (n, %)	453 (82.07) 127 (23.01) 105 (19.02)	118 (77.63) 49 (32.24) 35 (23.03)	335 (83.75) 78 (19.50) 70 (17.50)	0.094 0.001 0.139
Chronic lung disease (n, %)	81 (14.67)	27 (17.76)	54 (13.50)	0.206
Chronic kidney disease (n, %)	257 (46.56)	72 (47.37)	185 (46.25)	0.814
ESKD with HD (n, %)	74 (13.41)	22 (14.47)	52 (13.00)	0.650
Cirrhosis (n, %)	41 (7.43)	15 (9.87)	26 (6.50)	0.178
Metastatic cancer (n, %)	138 (25.00)	22 (14.47)	116 (29.00)	< 0.001
Neurological disease (n, %) - Old CVA (n, %) - Parkinson disease (n, %) - Alzhiemer’s disease or dementia (n, %)	301 (54.53) 238 (43.12) 21 (3.80) 26(4.71)	88 (57.89 47 (30.92) 11 (7.24) 15 (9.87)	213 (53.25 191 (47.75) 10 (2.50) 11 (2.75)	0.328 < 0.001 0.009 < 0.001
Hematological malignancy and on immunosuppressive drug (n, %)	25 (4.53)	8 (5.26)	17 (4.25)	0.609
AIDS (n, %)	7 (1.27)	2 (1.32)	5 (1.25)	0.951
**Source of infection**				
Lower respiratory tract infection (n, %)	175 (31.70)	43 (28.29)	132 (33.00)	0.288
Genitourinary tract infection (n, %)	229 (41.49)	67 (44.08)	162 (40.50)	0.446
Cardiovascular system infection (n, %)	5 (0.91)	1 (0.66)	4 (1.00)	0.705
Catheter-related bloodstream infection (n, %)	34 (6.16)	9 (5.92)	25 (6.25)	0.886
Skin, soft tissue and musculoskeletal infection (n, %)	34 (6.16)	9 (5.92)	25 (6.25)	0.886
Hepatobiliary tract infection (n, %)	26 (4.71)	6 (3.95)	20 (5.00)	0.602
Gastrointestinal infection (n, %)	36 (6.52)	12 (7.89)	24 (6.00)	0.421
Central nervous system infection (n, %)	13 (2.36)	5 (3.29)	8 (2.00)	0.372
**Physical examinations**				
Temperature, celsius (mean ± SD)	37.90 ± 1.15	37.85 ± 1.26	37.91 ± 1.10	0.601
HR, beats per minute (median, IQR)	100 (86, 118)	102 (85, 120)	100 (86, 117)	0.543
RR ≥ 22/minute (n, %)	420 (76.09)	122 (80.26)	298 (74.50)	0.156
SBP, mmHg (median, IQR)	125 (101, 148)	99 (83.5, 116)	133 (113, 155)	< 0.001
DBP, mmHg (median, IQR)	67 (57, 79)	58 (50.5, 67)	71 (62, 80)	< 0.001
MAP, mmHg (mean ± SD)	88.52 ± 21.82	75.38 ± 21.43	93.51 ± 19.82	< 0.001
SI, (mean ± SD)	0.86 ± 0.33	1.06 ± 0.40	0.79 ± 0.27	< 0.001
SpO_2_ (median, IQR)	94 (93, 96)	94 (92, 96)	95 (93, 96)	< 0.001
GCS (median, IQR)	15 (12, 15)	14 (10, 15)	15 (12.5, 15)	< 0.001
Urine output, ml/kg/hour, median (IQR)	1.06 (0.64, 1.53)	0.89 (0.49, 1.42)	1.14 (0.71, 1.57)	0.023
**Laboratory parameters**				
WBC, cells/mm^3^, (median, IQR)	12,085 (8515, 16345)	12,270 (8645, 17415)	11,925 (8450, 15855)	0.567
Platelet count, x10^3^/mm^3^ (median, IQR)	226.50 (156.00, 300.00)	195.00 (125.50, 255.50)	237.50 (173.50, 310.00)	0.001
POCT-glucose, mg/dL (median, IQR)	168.50 (170.00, 160.00)	160.00 (107.00, 231.00)	170.00 (124.00, 232.00)	0.299
BUN, mg/dL (median, IQR)	26 (17, 44)	31 (21, 48)	24 (15, 43)	0.003
Cr, mg/dL (median, IQR)	1.30 (0.84, 2.31)	1.66 (1.04, 3.06)	1.14 (0.81, 1.96)	< 0.001
Na, mmol/L (mean ± SD)	135.40 ± 7.11	135.66 ± 8.00	135.30 ± 6.78	0.601
K, mmol/L (mean ± SD)	4.26 ± 0.80	4.25 ± 0.85	4.27 ± 0.78	0.866
HCO_3_^−^ , mmol/L (mean ± SD)	19.75 ± 4.58	18.71 ± 4.96	20.14 ± 4.36	0.001
Bilirubin, mg/dL (median, IQR)	0.70 (0.50, 1.30)	0.70 (0.50, 1.30)	0.70 (0.50, 1.10)	0.608
Albumin, mg/dL (mean ± SD)	2.83 ± 0.67	2.62 ± 0.63	2.91 ± 0.66	< 0.001
pH (mean ± SD)	7.41 ± 0.08	7.39 ± 0.10	7.42 ± 0.08	< 0.001
PF ratio (mean ± SD)	362.95 ± 157.24	358.97 ± 183.04	364.46 ± 146.48	0.715
Arterial lactate, mmol/L (median, IQR)	1.82 (1.10, 3.20)	2.62 (1.59, 4.25)	1.66 (0.91, 2.63)	< 0.001
Arterial lactate > 2 mmol/L (n, %)	246 (44.57)	96 (63.16)	150 (37.5)	< 0.001
**Triage**				
ESI level 1–2 (n, %)	462 (83.70)	142 (93.42)	320 (80.00)	< 0.001
ESI level 3–5 (n, %)	90 (16.30)	10 (6.58)	80 (20.00)	< 0.001
qSOFA (median, IQR)	1 (1, 2)	2 (1, 3)	1 (1, 2)	< 0.001
SIRS (median, IQR)	3 (2, 3)	3 (2, 3)	2 (2, 3)	< 0.001
REWS (median, IQR)	4 (2, 6)	5 (4, 7)	3 (2, 5)	< 0.001
MEWS score (median, IQR)	6 (4, 7)	7 (5, 8)	5 (4, 7)	< 0.001
SAPS II score (median, IQR)	38 (31, 48)	47.5 (35, 57)	36 (30, 44)	< 0.001
SOFA score (median, IQR)	4 (3, 7)	8 (7, 10)	3 (2, 5)	< 0.001

Data are presented as mean ± standard deviation, frequency (%), and median (inter quartile range), BMI: body mass index, HbA1C: hemoglobin A1c, EMS: emergency medical service, NYHA: New York Heart Association, ESKD: end stage kidney disease, HD: hemodialysis, AIDS: Acquired immunodeficiency syndrome, HR: heart rate, RR: respiratory rate, h: hours, SBP: Systolic blood pressure, DBP: diastolic blood pressure, RR: Respiratory rate, MAP: mean arterial blood pressure, SI: shock index, SpO2: peripheral oxygen saturation, GCS: Glasgow Coma Scale, WBC: White blood cell, POCT glucose: point-of-care-testing blood glucose, BUN: Blood urea nitrogen, Cr: Creatinine, Na: sodium, K: potassium, HCO3-: bicarbonate, PF ratio: ratio of partial pressure of oxygen in arterial blood to the fraction of inspired oxygen, ESI: emergency severity index, qSOFA: quick Sequential Organ Failure Assessment Score, SOFA: quick Sequential Organ Failure Assessment Score, MEWS: Modified Early Warning Score, SIRS: systemic inflammatory response syndrome, SAPS II: Simplified Acute Physiology Score, REWS: Ramathibodi Early Warning Score and APACHE II: Acute Physiology and Chronic Health Evaluation

There were no significant differences in the mean age, body mass index (BMI) > 30 kg/m^2^, mean hemoglobin A1C (HbA1C) value, poorly controlled DM patients, Emergency Medical Service (EMS) route, surgical service, or the source of infection between the two groups. The genitourinary tract infection and lower respiratory tract infection were the first and second most common sources of infection in both groups. Physical examinations and laboratory parameters showed that systolic blood pressure (SBP), diastolic blood pressure (DBP), the shock index (SI), peripheral oxygen saturation (SpO2), Glasgow Coma scale (GCS), urine output, blood urea nitrogen (BUN), creatinine (Cr), bicarbonate (HCO_3_^−^) values, serum albumin, pH, and lactate concentrations were significantly different between two groups (all *p* < 0.05). The median scores for triage parameters: Emergency Severity Index (ESI), qSOFA, SIRS, REWS, MEWS, SAPS II, SOFA and APACHE II significantly differed between two groups (all *p* < 0.001).

Table 2 reveals that patients with septic shock had a significantly more extended hospital stay than those in the sepsis group (*p* < 0.001). We found a significantly higher rate of admission to the intensive care unit (ICU) in the septic shock group than in the sepsis group (*p* < 0.001). Moreover, the in-hospital mortality rates was approximately 2% for patients with poorly controlled DM who did not progress to septic shock and 20% for those who did. In patients with well-controlled DM, the in-hospital mortality rate was approximately 3% for those who did not progress to septic shock and 15% for those who did. Mortality rates were significantly higher in the septic shock group compared to the sepsis group for both poorly controlled DM (*p* < 0.001) and well-controlled DM (*p* = 0.039). When lactate concentrations exceeded ≥ 2 mmol/L and ≥ 4 mmol/L, the in-hospital mortality rates were significantly higher in the septic shock group than in the sepsis group (22.88% vs. 2.67%, *p* < 0.001 and 36.59% vs. 6.52%, *p* = 0.001, respectively). The in-hospital mortality rates, when antibiotics were administered within 1 h and delayed longer than 1 h, were significantly higher in the septic shock group than in the sepsis group (both *p* < 0.001). The median length of stay in the ED in patients discharged from the ED was not significantly different between the groups.

**Table 2:** Outcomes of diabetes patients with sepsis categorized by shock

Patient outcomes	All sepsis (*n* = 552)	Progress to Septic Shock (*n* = 152)	Not progress to septic shock (*n* = 400)	*p*-value
Time from ED to septic shock onset, minutes (median, IQR)	249.50 (137.50, 448.50)	249.50 (137.50, 448.50)	-	-
Time to antibiotic administration, minutes (median, IQR)	53.00 (39.00, 78.50)	53.50 (37.00, 79.00)	53.00 (40.00, 78.50)	0.779
Time to antibiotic administration more than 1 h (n, %)	223 (40.40)	62 (40.79)	161 (40.25)	0.908
Admission to the ICU (n, %)	181 (32.79)	101 (66.45)	80 (20.00)	< 0.001
Discharge from the ED (n, %)	144 (26.09)	16 (10.53)	128 (32.00)	< 0.001
ED-LOS with discharge from the ED, hours (median, IQR)	50.50 (18.00, 77.00)	51.00 (31.50, 78.00)	50.50 (15.50, 77.00)	0.952
Length of stay in the ED, hours (median, IQR)	19.50 (7.00, 49.00)	11.00 (6.00, 22.50)	26.00 (9.00, 59.00)	< 0.001
Length of stay in the hospital, days (median, IQR)	8.56 (3.85, 16.50)	11.94 (6.02, 18.92)	7.58 (3.06, 14.23)	< 0.001
Length of stay in the hospital with POCT-glucose ≥ 200 mg/dL, days (median, IQR)	8.08 (3.21, 17.29)	9.29 (4.75, 18.21)	7.90 (3.12, 15.88)	0.488
Length of stay in the hospital with POCT-glucose ≤ 70 mg/dL, days (median, IQR)	9.38 (5.13, 28.00)	10.35 (6.54, 28.00)	5.125 (1.33, 34.79)	0.643
Mortality within 28 days after ED visit (n, %)	32 (5.80)	24 (15.79)	8 (2.00)	< 0.001
In-hospital mortality (n, %)	40 (7.25)	29 (19.08)	11 (2.75)	< 0.001
In-hospital mortality with well controlled DM (n, %)	7 (7.78)	5 (15.63)	2 (3.45)	0.039
In-hospital mortality with poorly controlled DM (n, %)	33 (7.14)	24 (20)	9 (2.63)	< 0.001
In-hospital mortality with antibiotic administration within 1 h (n, %)	22 (3.99)	15 (9.87)	7 (1.75)	< 0.001
In-hospital mortality with a delay in antibiotic administration for > 1 h (n, %)	18 (3.26)	14 (9.21)	4 (1.00)	< 0.001
In-hospital mortality with lactate concentrations > 2 mmol/L (n, %)	25 (10.16)	21 (21.88)	4 (2.67)	< 0.001
In-hospital mortality with lactate concentrations > 4 mmol/L (n, %)	18 (20.69)	15 (36.59)	3 (6.52)	0.001
Mechanical ventilation used (n, %) - Endotracheal tube (n, %) - Non-invasive ventilator (n, %)	226 (40.94) 143 (25.91) 83 (15.04)	99 (65.13) 71 (46.71) 28 (18.42)	127 (31.75) 72 (18.00) 55 (13.75)	< 0.001 < 0.001 0.170

Data are presented as mean ± standard deviation, frequency (%), and median (inter quartile range), ICU: intensive care unit, LOS: length of stay, ED-LOS: emergency department length of stay, h: hours and POCT glucose: point-of-care-testing blood glucose

**Fig. 2:**
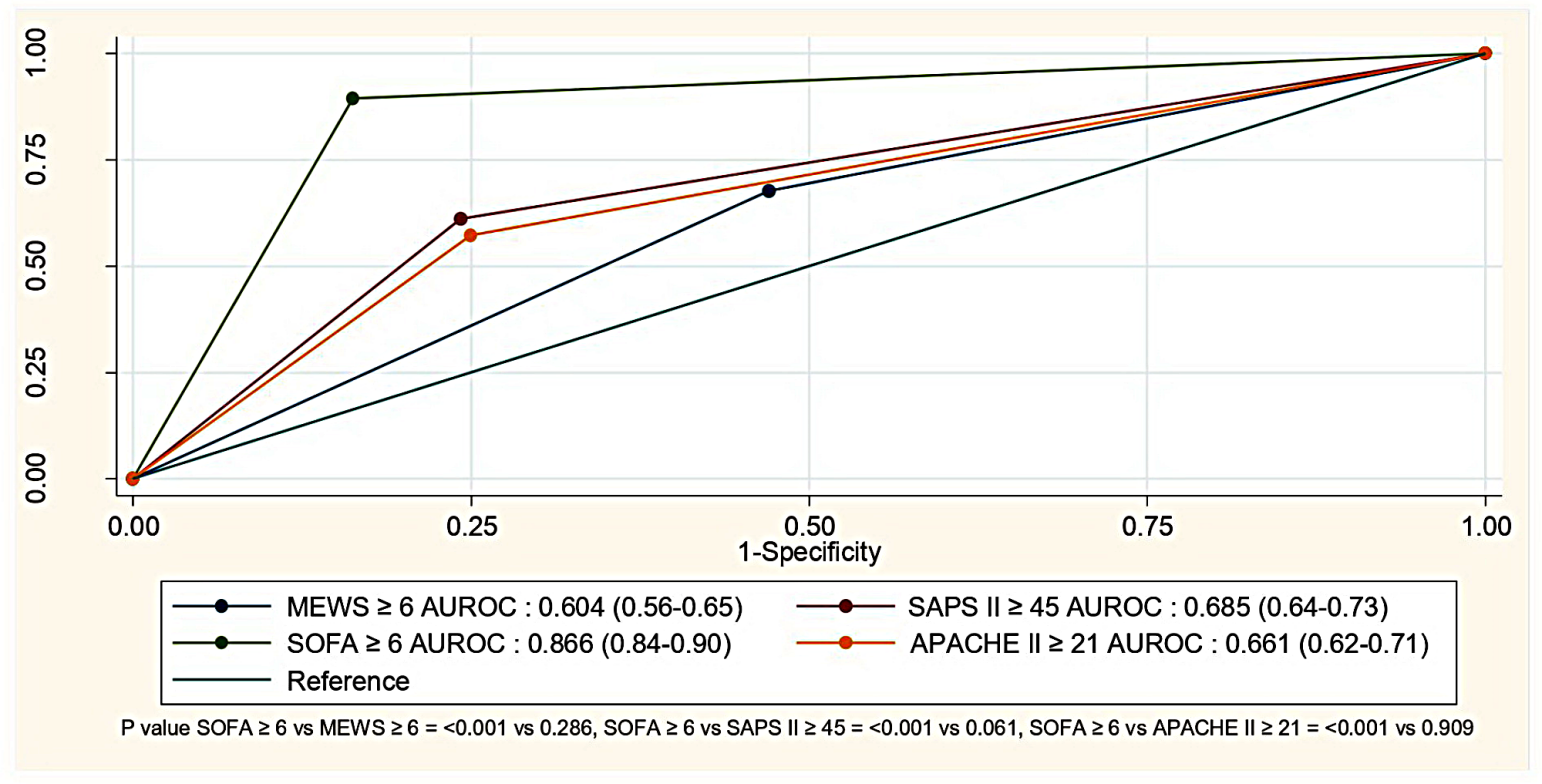
Diagnostic accuracy of MEWS, SAPS II, SOFA, and APACHE II scores to predict shock in patients with diabetes within 48 h after visiting the ED

**Fig. 3:**
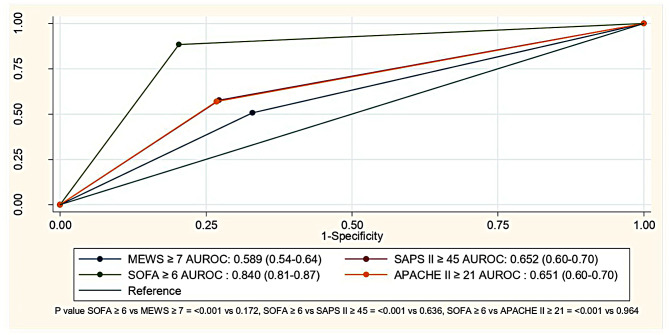
Diagnostic accuracy of MEWS, SAPS II, SOFA, and APACHE II scores to predict shock in patients with diabetes 2 h after an ED visit (*n* = 130)

Table 3 shows the predictive performances of scores for septic shock onset within 48 h using the area under the Receiver operating characteristic (AUROC) curve. Notably, a SOFA score ≥ 6 (supplementary Table 1) showed robust discrimination at 0.866 (95% confidence interval [CI] 0.84–0.90, *p* < 0.001), with an optimal cutoff of 5.5 points and a maximum Youden index of 0.732. In contrast, MEWS, SAPS II, and APACHE II scores lacked effective discrimination. The MEWS score (cutoff: 5.5 points) had a Youden index of 0.208, the SAPS II score (cutoff: 44.5 points) had a Youden index of 0.369, and the APACHE II score (cutoff: 20.5 points) had a Youden index of 0.322. Their AUROC values were 0.647 (95% CI 0.60–0.70), 0.707 (95% CI 0.66–0.76), and 0.668 (95% CI 0.61–0.72), respectively. These results are shown in Fig. 2 and supplementary Fig. 1 for predicted shock 48 h post-ED visit. Additionally, the score predicting shock after a 2-hour ED visit was significantly good for a SOFA score ≥ 6 (AUROC = 0.840, 95% CI 0.81–0.87, *p* < 0.001), but the other scores showed insufficient discriminatory power (Fig. 3, supplementary Table *2*, supplementary Figs. 2–3). A subgroup analysis for well controlled and poorly controlled DM identified the SOFA score as the best predictor, with AUROCs of 0.938 (95% CI 0.91–0.96) and 0.941 (95% CI 0.90–0.99), respectively (supplementary Table 3). Multivariable logistic regression analysis for septic shock showed significant results for SBP, SpO2, GCS, pH, and lactate concentrations (r^2^ = 0.25, all *p* < 0.05, supplementary Table 4).

**Table 3:** Diagnostic accuracy of MEWS, SAPS II, SOFA, and APACHE II scores to predict shock in patients with diabetes within 48 h after visiting the ED

Score	Cutoff	Odds ratio	Sens (95% CI)	Spec (95% CI)	PPV (95% CI)	NPV (95% CI)	LR+ (95% CI)	LR- (95% CI)	AUROC (95% CI)	*p*-value*
**SOFA**	≥ 6	43.81 (24.59–77.98)	89.5% (83.5–93.9%)	83.8% (79.8–87.2%)	67.7% (60.7–74.1%)	95.4% (92.7–97.4%)	5.51 (4.38–6.92)	0.13 (0.08–0.20)	0.866 (0.84–0.90)	-
**MEWS**	≥ 6	2.37 (1.60–3.51)	67.8% (59.7–75.1%)	53.0% (48.0–58.0%)	35.4% (29.9–41.2%)	81.2% (76-85.8%)	1.44 (1.24–1.68)	0.61 (0.47–0.78)	0.604 (0.56–0.65)	0.286
**SAPS II**	≥ 45	4.92 (3.31–7.33)	61.2% (53.0–69.0%)	75.8% (71.2–79.9%)	48.9% (41.6–56.3%)	83.7% (79.5–87.4%)	2.52 (2.04–3.13)	0.51 (0.42–0.63)	0.685 (0.64–0.73)	0.061
**APACHE II**	≥ 21	4.02 (2.71–5.94)	57.2% (49.0-65.2%)	75% (70.5–79.2%)	46.5% (39.2–53.9%)	82.2% (77.9–86.0%)	2.29 (1.84–2.85)	0.57 (0.47–0.69)	0.661 (0.62–0.71)	0.909

* P valued compared with SOFA ≥ 6 (*p* < 0.001)

Data are presented as odds ratio 95% CI: confidence Interval, SD: standard deviation, AUROC: Area under the ROC curve, Sens: sensitivity, Spec: Specificity, PPV: positive predictive values, NPV: negative predictive values and LR: likelihood ratio

## Discussion

In our study, patients in the sepsis group had a mean age of 76.43 years, and those in the septic shock group had a mean age of 74.27 years, with no significant difference between them. These findings are consistent with a study by Esposito et al. These findings suggest an increased risk of sepsis in individuals aged older than 60 years. Additionally, this study showed an association between male sex and septic shock, suggesting that male sex hormones impair cell-mediated immune responses. [17]

This study shows nearly 40% of the patients (from both cohorts) did not receive antibiotics within 1 h and almost 10% of septic shock patients were discharged from ED. Early prediction of septic shock could be utilized in these cases and hopefully alter the mortality. There were 66.45% of patients with septic shock admitted to the ICU, while the remainder discontinued vasopressor/inotropic drugs in the ED, awaiting ICU admission. Notably, patients with sepsis showed a higher direct discharge rate from the ED than those with septic shock. Additionally, there was no significant difference in the length of stay of patients discharged from the ED between the two groups. The median time of ED-LOS with discharge from ED in patients with sepsis was 50.5 h (IQR; 15.5, 77) and in septic shock group was 51 h (IQR; 31.5, 78). Alternatively, this finding could have been limited by our study’s single-center design and small sample size, especially compared with a study by Calvert et al. on 29,083 patients who progressed to septic shock. [18] In our study, the in-hospital mortality rate of septic shock group was 19.08%, consistent with Wardi et al.’s study. [19] Poorly controlled diabetes or point-of-care-testing glucose; ≥ 200, < 70 mg/dL, did not show a correlation with the overall mortality rate. However, this study shows the mortality rate for patients with poorly controlled diabetes was ten times higher than that for patients with sepsis who developed septic shock. In contrast, the mortality rate for patients with well controlled diabetes was five times higher compared to these groups. Costantini et al. suggested ongoing controversy regarding the effect of diabetes on sepsis mortality. [4] The high mortality rates in diabetic patients with sepsis and septic shock underscore the need for early recognition, prompt treatment, and tight glycemic control in these high-risk individuals. Furthermore, optimizing diabetes management is crucial to minimizing infection risk and severity. Notably, in our study, metastatic cancer cases were significantly more prevalent in patients with sepsis than in those with septic shock. This difference between studies may be due to the exclusion of palliative care in septic shock cases, affecting SAPS II and APACHE II scores.

We found that SBP, SpO2, GCS, pH, and lactate concentrations were predictors of septic shock, which is consistent with the findings of Wardi et al., who have utilized these parameters to predict septic shock, particularly in emergency settings. These parameters serve as critical indicators of patient status and can stratify patients based on their risk for severe outcomes, thereby guiding clinical decisions regarding the level of care required, such as ICU admission. Notably, various triage scores (ESI, qSOFA, SIRS, REWS, MEWS, SOFA, SAPS II, and APACHE II) were significantly different between patients with sepsis and those with septic shock, which suggested their usefulness in predicting the severity of sepsis. [19]

This study showed that the SOFA score was the optimal predictor in predicting septic shock, with a cutoff point at ≥ 6 and an impressive AUROC range of 0.8–0.9 within 48 h post-ED visit and within 2 h for patients with diabetes. When diagnosing septic shock, physicians must consider the infection source, hypotension requiring vasopressors to maintain mean arterial pressure > 65 mmHg, and adequate fluid resuscitation (evaluated by clinical and ultrasound criteria). Additionally, recognizing the operator-dependent nature of ultrasound is essential. Importantly, in our study, the operators (most were residents trained in emergency medicine) obtained approval through resuscitation and ultrasonography courses.

MEWS, SAPS II, SOFA, and APACHE II scores involve SBP. The SOFA score, which integrates values related to vasopressor/inotropic drug use and mean arterial pressure, remains relevant, even though these values were collected in our study before the onset of septic shock. Based on these factors, we recommend using the SOFA score with a cutoff point of ≥ 6 because of its high sensitivity and specificity in predicting septic shock. Regular monitoring of this score could facilitate the early identification of septic shock within 48 h and, notably, as early as 2 h post-ED visit.

## Conclusion

SBP, SpO2, GCS, pH, and lactate concentrations are crucial for the early prediction of septic shock in patients with diabetes. The SOFA score is a superior predictor for the onset of septic shock in patients with diabetes compared with MEWS, SAPS II, and APACHE II scores. Specifically, a cutoff of ≥ 6 in the SOFA score demonstrates high accuracy in predicting shock within 48 h post-ED visit and as early as 2 h after ED admission.

## Limitations

A study at a specific tertiary care hospital may not fully represent the broader population because of given the generally less complex nature of patients in rural hospitals. Therefore, it is unclear whether the patient characteristics, treatment protocols, and outcomes would be representative of diabetic patients presenting with sepsis at other institutions or in different geographic regions. The limited sample size, falling short of 693 events, could affect our results, especially non-statistical outcomes. This influence effect could be mitigated through an increase in sample size. Diagnosing septic shock involves clinical and operator-dependent ultrasound assessments, contributing to the overall complexity of the diagnosis. For improvement, data collection mandates approval from operators with completed resuscitation and ultrasonography courses, enhancing methodological rigor.

## Electronic supplementary material

Below is the link to the electronic supplementary material.

Supplementary Material 1

Supplementary Material 2

Supplementary Material 3

Supplementary Material 4

Supplementary Material 5

Supplementary Material 6

Supplementary Material 7

## Data Availability

The datasets used and/ or analyzed during the current study are available from the corresponding author upon reasonable request. Additional data is provided within supplementary information files.

## Declarations

### Use of AI

During the preparation of this work, the authors used ChatGPT and Grammarly to refine and modify the language. After using this application, the authors reviewed and edited the content as required and took full responsibility for the manuscript’s content.
