# Supplementary material for: Comparison of Modified Early Warning Score (MEWS), Simplified Acute Physiology Score II (SAPS II), Sequential Organ Failure Assessment (SOFA), and Acute Physiology and Chronic Health Evaluation II (APACHE II) for early prediction of septic shock in diabetic patients in Emergency Departments
Source: BMC Emerg Med. 2024 Sep 4;24:161. doi: 10.1186/s12873-024-01078-8 (PMC11376032; doi:10.1186/s12873-024-01078-8)
Supplement: Supplementary file 3 — Supplementary Material 3 [file 12873_2024_1078_MOESM3_ESM.docx]

**Supplementary Figure 3** Diagnostic accuracy of MEWS, SAPS II, SOFA, and APACHE II scores to predict shock in patients with poorly controlled (n=90) diabetes
