# Supplementary material for: Comparison of Modified Early Warning Score (MEWS), Simplified Acute Physiology Score II (SAPS II), Sequential Organ Failure Assessment (SOFA), and Acute Physiology and Chronic Health Evaluation II (APACHE II) for early prediction of septic shock in diabetic patients in Emergency Departments
Source: BMC Emerg Med. 2024 Sep 4;24:161. doi: 10.1186/s12873-024-01078-8 (PMC11376032; doi:10.1186/s12873-024-01078-8)
Supplement: Supplementary file 4 — Supplementary Material 4 [file 12873_2024_1078_MOESM4_ESM.docx]

**Supplementary Table 1 : Cut point for MEWS, SAPS II, SOFA, and APACHE II**

**Supplementary Table 1.1** Cut point for MEWS

Detailed report of sensitivity and specificity

Correctly

Cut point Sensitivity Specificity Classified LR+ LR-

( >= 1 ) 100.00% 0.00% 27.54% 1.0000

( >= 2 ) 99.34% 2.75% 29.35% 1.0215 0.2392

( >= 3 ) 97.37% 9.50% 33.70% 1.0759 0.2770

( >= 4 ) 89.47% 24.50% 42.39% 1.1851 0.4296

( >= 5 ) 80.26% 36.25% 48.37% 1.2590 0.5445

( >= 6 ) 67.76% 53.00% 57.07% 1.4418 0.6082

( >= 7 ) 50.66% 68.00% 63.22% 1.5831 0.7256

( >= 8 ) 35.53% 82.25% 69.38% 2.0015 0.7839

( >= 9 ) 23.03% 92.00% 73.01% 2.8783 0.8367

( >= 10 ) 11.84% 96.00% 72.83% 2.9605 0.9183

( >= 11 ) 7.24% 98.75% 73.55% 5.7895 0.9394

( >= 12 ) 3.95% 99.75% 73.37% 15.7895 0.9629

( >= 13 ) 2.63% 100.00% 73.19% 0.9737

( >= 14 ) 1.32% 100.00% 72.83% 0.9868

( > 14 ) 0.00% 100.00% 72.46% 1.0000

ROC -Asymptotic Normal--

Obs Area Std. Err. [95% Conf. Interval]

------------------------------------------------------------

552 0.6466 0.0259 0.59579 0.69747

**Empirical cutpoint estimation for MEWS**

Empirical optimal cutpoint: 5.5

Youden index (J): 0.208

SE(J): 0.0454

Sensitivity at cutpoint: 0.68

Specificity at cutpoint: 0.53

Area under ROC curve at cutpoint: 0.60

**Supplementary Table 1.2** Cut point for SAPS II

Detailed report of sensitivity and specificity

Correctly

Cutpoint Sensitivity Specificity Classified LR+ LR-

( >= 12 ) 100.00% 0.00% 27.54% 1.0000

( >= 13 ) 100.00% 0.50% 27.90% 1.0050 0.0000

( >= 15 ) 100.00% 0.75% 28.08% 1.0076 0.0000

( >= 16 ) 100.00% 1.25% 28.44% 1.0127 0.0000

( >= 17 ) 100.00% 1.50% 28.62% 1.0152 0.0000

( >= 18 ) 100.00% 2.00% 28.99% 1.0204 0.0000

( >= 19 ) 99.34% 2.75% 29.35% 1.0215 0.2392

( >= 20 ) 99.34% 3.00% 29.53% 1.0241 0.2193

( >= 21 ) 99.34% 3.50% 29.89% 1.0295 0.1880

( >= 22 ) 99.34% 5.00% 30.98% 1.0457 0.1316

( >= 23 ) 98.68% 6.50% 31.88% 1.0554 0.2024

( >= 24 ) 98.68% 7.75% 32.79% 1.0697 0.1698

( >= 25 ) 95.39% 10.00% 33.51% 1.0599 0.4605

( >= 26 ) 94.74% 11.25% 34.24% 1.0675 0.4678

( >= 27 ) 94.74% 14.50% 36.59% 1.1080 0.3630

( >= 28 ) 92.76% 16.00% 37.14% 1.1043 0.4523

( >= 29 ) 92.11% 18.75% 38.95% 1.1336 0.4211

( >= 30 ) 88.16% 22.50% 40.58% 1.1375 0.5263

( >= 31 ) 87.50% 29.25% 45.29% 1.2367 0.4274

( >= 32 ) 85.53% 33.75% 48.01% 1.2910 0.4289

( >= 33 ) 82.89% 35.75% 48.73% 1.2902 0.4785

( >= 34 ) 78.95% 40.75% 51.27% 1.3324 0.5166

( >= 35 ) 77.63% 45.50% 54.35% 1.4244 0.4916

( >= 36 ) 73.68% 48.25% 55.25% 1.4238 0.5454

( >= 37 ) 69.74% 52.00% 56.88% 1.4529 0.5820

( >= 38 ) 67.11% 55.75% 58.88% 1.5165 0.5900

Cutpoint Sensitivity Specificity Classified LR+ LR-

( >= 39 ) 67.11% 57.75% 60.33% 1.5883 0.5696

( >= 40 ) 67.11% 62.25% 63.59% 1.7776 0.5284

( >= 41 ) 66.45% 63.75% 64.49% 1.8330 0.5263

( >= 42 ) 65.13% 67.50% 66.85% 2.0040 0.5166

( >= 43 ) 64.47% 71.00% 69.20% 2.2232 0.5004

( >= 44 ) 62.50% 73.75% 70.65% 2.3810 0.5085

( >= 45 ) 61.18% 75.75% 71.74% 2.5231 0.5124

( >= 46 ) 56.58% 78.25% 72.28% 2.6013 0.5549

( >= 47 ) 53.95% 79.50% 72.46% 2.6316 0.5793

( >= 48 ) 50.00% 83.75% 74.46% 3.0769 0.5970

( >= 49 ) 48.03% 85.50% 75.18% 3.3122 0.6079

( >= 50 ) 43.42% 87.50% 75.36% 3.4737 0.6466

( >= 51 ) 38.82% 89.00% 75.18% 3.5287 0.6875

( >= 52 ) 36.84% 90.25% 75.54% 3.7787 0.6998

( >= 53 ) 34.87% 92.50% 76.63% 4.6491 0.7041

( >= 54 ) 32.89% 93.75% 76.99% 5.2632 0.7158

( >= 55 ) 30.92% 94.50% 76.99% 5.6220 0.7310

( >= 56 ) 29.61% 94.75% 76.81% 5.6391 0.7430

( >= 57 ) 26.32% 95.25% 76.27% 5.5402 0.7736

( >= 58 ) 23.03% 95.50% 75.54% 5.1170 0.8060

( >= 59 ) 20.39% 95.50% 74.82% 4.5322 0.8336

( >= 60 ) 19.74% 96.25% 75.18% 5.2632 0.8339

( >= 61 ) 17.11% 96.25% 74.46% 4.5614 0.8612

( >= 62 ) 17.11% 96.50% 74.64% 4.8872 0.8590

( >= 63 ) 16.45% 97.00% 74.82% 5.4825 0.8614

( >= 64 ) 15.13% 97.25% 74.64% 5.5024 0.8727

( >= 65 ) 13.82% 98.00% 74.82% 6.9079 0.8794

( >= 66 ) 11.84% 98.25% 74.46% 6.7669 0.8973

( >= 68 ) 11.18% 98.25% 74.28% 6.3910 0.9040

Cutpoint Sensitivity Specificity Classified LR+ LR-

( >= 69 ) 9.21% 98.50% 73.91% 6.1404 0.9217

( >= 70 ) 8.55% 98.50% 73.73% 5.7018 0.9284

( >= 71 ) 7.89% 98.75% 73.73% 6.3158 0.9327

( >= 72 ) 7.24% 98.75% 73.55% 5.7895 0.9394

( >= 75 ) 5.92% 99.00% 73.37% 5.9211 0.9503

( >= 76 ) 5.26% 99.00% 73.19% 5.2632 0.9569

( >= 77 ) 5.26% 99.25% 73.37% 7.0175 0.9545

( >= 78 ) 3.29% 99.25% 72.83% 4.3860 0.9744

( >= 79 ) 2.63% 99.50% 72.83% 5.2632 0.9786

( >= 80 ) 1.97% 99.50% 72.64% 3.9474 0.9852

( >= 81 ) 1.32% 99.50% 72.46% 2.6316 0.9918

( >= 83 ) 0.00% 99.50% 72.10% 0.0000 1.0050

( >= 96 ) 0.00% 99.75% 72.28% 0.0000 1.0025

( > 96 ) 0.00% 100.00% 72.46% 1.0000

ROC -Asymptotic Normal--

Obs Area Std. Err. [95% Conf. Interval]

------------------------------------------------------------

552 0.7069 0.0262 0.65545 0.75831

**Empirical cutpoint estimation for SAPS II**

Empirical optimal cutpoint: 44.5

Youden index (J): 0.369

SE(J): 0.0450

Sensitivity at cutpoint: 0.61

Specificity at cutpoint: 0.76

Area under ROC curve at cutpoint: 0.68

**Supplementary Table 1.3** Cut point for SOFA

Detailed report of sensitivity and specificity

Correctly

Cutpoint Sensitivity Specificity Classified LR+ LR-

( >= 0 ) 100.00% 0.00% 27.54% 1.0000

( >= 1 ) 100.00% 3.25% 29.89% 1.0336 0.0000

( >= 2 ) 100.00% 12.00% 36.23% 1.1364 0.0000

( >= 3 ) 100.00% 33.50% 51.81% 1.5038 0.0000

( >= 4 ) 98.68% 53.75% 66.12% 2.1337 0.0245

( >= 5 ) 94.74% 68.75% 75.91% 3.0316 0.0766

( >= 6 ) 89.47% 83.75% 85.33% 5.5061 0.1257

( >= 7 ) 78.95% 93.50% 89.49% 12.1457 0.2252

( >= 8 ) 62.50% 96.75% 87.32% 19.2308 0.3876

( >= 9 ) 47.37% 98.25% 84.24% 27.0677 0.5357

( >= 10 ) 33.55% 98.75% 80.80% 26.8421 0.6729

( >= 11 ) 19.74% 99.25% 77.36% 26.3158 0.8087

( >= 12 ) 10.53% 99.75% 75.18% 42.1053 0.8970

( >= 13 ) 4.61% 100.00% 73.73% 0.9539

( >= 15 ) 0.66% 100.00% 72.64% 0.9934

( > 15 ) 0.00% 100.00% 72.46% 1.0000

ROC -Asymptotic Normal--

Obs Area Std. Err. [95% Conf. Interval]

------------------------------------------------------------

552 0.9370 0.0106 0.91628 0.95776

**Empirical cutpoint estimation for SOFA**

Empirical optimal cutpoint: 5.5

Youden index (J): 0.732

SE(J): 0.0310

Sensitivity at cutpoint: 0.89

Specificity at cutpoint: 0.84

Area under ROC curve at cutpoint: 0.87

**Supplementary Table 1.4** Cut point for APACHE II

Detailed report of sensitivity and specificity

Correctly

Cutpoint Sensitivity Specificity Classified LR+ LR-

( >= 2 ) 100.00% 0.00% 27.54% 1.0000

( >= 4 ) 99.34% 0.25% 27.54% 0.9959 2.6316

( >= 6 ) 99.34% 0.50% 27.72% 0.9984 1.3158

( >= 7 ) 99.34% 1.50% 28.44% 1.0085 0.4386

( >= 8 ) 98.68% 3.25% 29.53% 1.0200 0.4049

( >= 9 ) 96.05% 6.00% 30.80% 1.0218 0.6579

( >= 10 ) 94.74% 8.50% 32.25% 1.0354 0.6192

( >= 11 ) 93.42% 11.50% 34.06% 1.0556 0.5721

( >= 12 ) 89.47% 15.75% 36.05% 1.0620 0.6683

( >= 13 ) 88.16% 19.50% 38.41% 1.0951 0.6073

( >= 14 ) 84.21% 24.50% 40.94% 1.1154 0.6445

( >= 15 ) 78.95% 31.75% 44.75% 1.1567 0.6631

( >= 16 ) 76.97% 40.75% 50.72% 1.2991 0.5651

( >= 17 ) 73.68% 46.75% 54.17% 1.3837 0.5629

( >= 18 ) 69.08% 54.50% 58.51% 1.5182 0.5674

( >= 19 ) 64.47% 61.75% 62.50% 1.6856 0.5753

( >= 20 ) 62.50% 67.75% 66.30% 1.9380 0.5535

( >= 21 ) 57.24% 75.00% 70.11% 2.2895 0.5702

( >= 22 ) 48.68% 78.75% 70.47% 2.2910 0.6516

( >= 23 ) 44.08% 81.50% 71.20% 2.3826 0.6861

( >= 24 ) 38.16% 84.50% 71.74% 2.4618 0.7319

( >= 25 ) 34.21% 87.25% 72.64% 2.6832 0.7540

( >= 26 ) 30.26% 90.25% 73.73% 3.1039 0.7727

( >= 27 ) 22.37% 93.50% 73.91% 3.4413 0.8303

( >= 28 ) 20.39% 94.50% 74.09% 3.7081 0.8424

( >= 29 ) 17.76% 96.25% 74.64% 4.7368 0.8544

( >= 30 ) 13.82% 97.00% 74.09% 4.6053 0.8885

( >= 31 ) 10.53% 97.75% 73.73% 4.6784 0.9153

( >= 32 ) 9.87% 98.25% 73.91% 5.6391 0.9174

( >= 33 ) 7.24% 98.50% 73.37% 4.8246 0.9418

( >= 34 ) 5.26% 99.00% 73.19% 5.2632 0.9569

( >= 35 ) 4.61% 99.00% 73.01% 4.6053 0.9636

( >= 36 ) 3.95% 99.50% 73.19% 7.8947 0.9654

( >= 39 ) 1.32% 99.50% 72.46% 2.6316 0.9918

( >= 41 ) 0.66% 99.50% 72.28% 1.3158 0.9984

( >= 42 ) 0.00% 99.75% 72.28% 0.0000 1.0025

( > 42 ) 0.00% 100.00% 72.46% 1.0000

ROC -Asymptotic Normal--

Obs Area Std. Err. [95% Conf. Interval]

------------------------------------------------------------

552 0.6680 0.0274 0.61436 0.72173

**Empirical cutpoint estimation for APACHE II**

Empirical optimal cutpoint: 20.5

Youden index (J): 0.322

SE(J): 0.0456

Sensitivity at cutpoint: 0.57

Specificity at cutpoint: 0.75

Area under ROC curve at cutpoint: 0.66
